# Sensorimotor dysfunction and postural instability in older adults with type 2 diabetes mellitus: the role of proprioception and neuropathy

**DOI:** 10.3389/fnagi.2025.1615399

**Published:** 2025-06-23

**Authors:** Saleh M. Kardm, Abdulmohsen Saeed Kardm, Ziad Ahmed Alanazi, Hani Hassan Alnakhli, Batool Abdulelah Alkhamis, Ravi Shankar Reddy

**Affiliations:** ^1^Department of Surgery, College of Medicine, Najran University, Najran, Saudi Arabia; ^2^Aseer Central Hospital, Abha, Saudi Arabia; ^3^Division of Orthopedic, Department of Surgery, College of Medicine, Jouf University, Sakaka, Saudi Arabia; ^4^Program of Physical Therapy, Department of Medical Rehabilitation Sciences, College of Applied Medical Sciences, King Khalid University, Abha, Saudi Arabia

**Keywords:** type 2 diabetes mellitus, proprioception, postural stability, peripheral neuropathy, glycemic control, posturography

## Abstract

**Background:**

Type 2 diabetes mellitus (T2DM) is associated with proprioceptive impairment and postural instability, contributing to increased fall risk. The role of glycemic status and peripheral neuropathy in these deficits remains under-characterized using objective measurement tools.

**Objectives:**

To compare ankle joint proprioception and postural stability between individuals with T2DM and healthy controls using dual inclinometry and computerized posturography, and to examine the relationship between these impairments and glycemic control (HbA1c). Additionally, to evaluate the impact of peripheral neuropathy on sensorimotor function.

**Methods:**

A cross-sectional study included 66 individuals with T2DM and 66 age- and sex-matched healthy controls. Ankle joint proprioception was assessed using dual digital inclinometers, which quantify joint position sense errors during dorsiflexion and plantarflexion. Postural stability was evaluated via posturography under eyes-closed conditions, measuring sway area, velocity, and center of pressure (CoP) displacement.

**Results:**

Participants with T2DM showed significantly greater proprioceptive errors in dorsiflexion (mean difference = 1.93°, 95% CI: 1.61–2.26, *d* = 2.06) and plantarflexion (mean difference = 2.50°, 95% CI: 2.08–2.92, *d* = 2.03) compared to controls. Postural sway area and velocity were also higher (sway area mean difference = 62.76 cm^2^, 95% CI: 47.44–78.07, *d* = 1.40). HbA1c levels were moderately correlated with proprioception errors (*r* ≈ 0.54) and postural instability (*r* ≈ 0.65). Participants with peripheral neuropathy demonstrated significantly worse proprioception and balance. Regression models showed HbA1c and diabetes duration were significantly associated with proprioceptive and postural impairments (adjusted *R*^2^: 0.29–0.48 for both domains).

**Conclusion:**

Individuals with T2DM, particularly those with poor glycemic control or peripheral neuropathy, show greater sensorimotor deficits. These findings support early proprioceptive screening and balance interventions to reduce fall risk in diabetic populations. All associations should be interpreted within the limitations of a cross-sectional design.

## Introduction

Type 2 diabetes mellitus (T2DM) is a chronic metabolic disorder characterized by insulin resistance, hyperglycemia, and progressive beta-cell dysfunction, affecting millions of individuals worldwide (Lima et al., 2022). Prolonged exposure to elevated blood glucose levels leads to systemic complications that impair multiple physiological functions, including neuromuscular control and postural stability (Ruze et al., 2023). Among the most prevalent complications of diabetes is peripheral neuropathy, a condition resulting from chronic hyperglycemia-induced nerve damage, affecting somatosensory feedback and motor coordination (Ruze et al., 2023). These deficits in sensory and motor control mechanisms have profound implications for functional mobility and fall risk, making postural instability a significant concern for individuals with T2DM (Kaur et al., 2023).

Proprioception, or joint position sense, is essential for maintaining postural control and dynamic stability, as it enables the integration of afferent signals from mechanoreceptors in the muscles, tendons, and joints (Tufvesson et al., 2023). Effective proprioceptive function ensures accurate motor responses, allowing individuals to maintain equilibrium and coordinated movements (Proske and Chen, 2021). In individuals with T2DM, diabetes-related nerve damage leads to altered proprioceptive feedback, contributing to poor postural control and impaired balance (González-Grandón et al., 2021). These impairments are particularly pronounced at the ankle joint, a key structure for maintaining postural adjustments during both static and dynamic tasks (Malwanage et al., 2024). Consequently, deficits in ankle joint proprioception may serve as a critical contributor to increased postural sway and fall susceptibility in this population (Reddy et al., 2024).

Postural stability is a multisensory process that depends on the effective coordination of the visual, vestibular, and somatosensory systems (Alshahrani et al., 2024). Individuals with T2DM, particularly those with peripheral neuropathy, exhibit increased postural sway, reduced balance confidence, and impaired gait patterns, which collectively increase the risk of falls and mobility limitations (Tele-Heri et al., 2021). Studies have shown that diabetes-related proprioceptive impairments lead to compensatory reliance on visual and vestibular feedback for balance control, which may be insufficient in challenging or low-visibility environments (Toloza-Cano et al., 2021; Enayati and Cacace, 2024). Moreover, glycemic dysregulation and long-term diabetes duration have been linked to progressive deterioration in postural stability, emphasizing the need for objective assessments of proprioceptive function and balance control in clinical evaluations of individuals with diabetes (Ahmad et al., 2023; Rasmussen et al., 2022).

Despite the well-established relationship between diabetes and postural instability, limited studies have quantitatively assessed proprioceptive deficits in individuals with T2DM using dual inclinometry (Ortiz et al., 2022; ALMohiza et al., 2023). They have not examined the direct impact of glycemic control on postural stability using posturography. Furthermore, while peripheral neuropathy is known to exacerbate sensorimotor dysfunction, there is a lack of comparative data evaluating proprioceptive and postural impairments between neuropathic and non-neuropathic diabetic individuals (Reeves et al., 2021). Additionally, glycemic control (HbA1c levels) as a predictor of proprioceptive deficits and balance impairments remains insufficiently explored. Moreover, unlike previous studies that focus predominantly on vestibular or visual components of balance, our study employed dual digital inclinometry to quantify directional ankle joint proprioceptive errors, and computerized posturography under eyes-closed conditions to isolate somatosensory-driven postural control. This methodological refinement allowed us to uncover proprioceptive-specific impairments in T2DM. Furthermore, the inclusion of subgroup analysis between neuropathic and non-neuropathic individuals and the integration of multivariate regression modeling to predict balance impairment based on metabolic and demographic variables represent substantial extensions to current knowledge. Understanding these relationships is crucial for developing targeted interventions to reduce fall risk and improve functional stability in individuals with T2DM. In this context, “early proprioceptive assessments” refer to the integration of joint position sense testing and balance evaluation at the initial stage of clinical functional assessment in patients with T2DM, regardless of disease duration. The aim is to identify proprioceptive decline proactively, before observable mobility loss or falls occur, thus facilitating timely intervention strategies within routine diabetic care. Given these gaps, this study aims to compare ankle joint proprioception and postural balance deficits between individuals with T2DM and healthy controls using dual inclinometry and posturography. Additionally, the study seeks to evaluate the relationship between proprioceptive impairments, postural instability, and glycemic control (HbA1c levels) while assessing differences between diabetic individuals with and without peripheral neuropathy.

## Materials and methods

### Design and ethics

This cross-sectional study was conducted between May 2023 and March 2024 at the Department of Endocrinology and Rehabilitation, Alfarah, a tertiary care center specializing in diabetes management and neuromuscular rehabilitation. Ethical approval was obtained from the Institutional Ethics Committee of KKU (REC# 245-2023), and the study adhered to the principles outlined in the Declaration of Helsinki. Written informed consent was obtained from all participants before enrollment.

### Participants

The T2DM group included individuals diagnosed based on the American Diabetes Association (ADA) 2023 criteria (Sloan et al., 2021), which defines T2DM as fasting plasma glucose (FPG) ≥ 126 mg/dL, glycated hemoglobin (HbA1c) ≥ 6.5%, or 2-h postprandial glucose ≥ 200 mg/dL following an oral glucose tolerance test (OGTT) (ElSayed et al., 2023). The healthy control group participants had no history of diabetes or metabolic disorders and exhibited normal fasting glucose and HbA1c levels. Individuals with T2DM were further subgrouped into neuropathic and non-neuropathic groups using the Toronto Clinical Scoring System (TCSS) and nerve conduction studies, with a TCSS score ≥ 6 indicating peripheral neuropathy.

Inclusion criteria for the T2DM group were: (1) confirmed diagnosis of T2DM for at least five years, (2) age between 40 and 65 years, (3) ability to stand independently for posturography testing, and (4) absence of acute diabetic complications at the time of assessment. The healthy control group was required to be age- and sex-matched, free from diabetes, and without any known neurological or musculoskeletal disorders. Exclusion criteria for all participants included (1) history of neurological disorders such as stroke, Parkinson’s disease, or multiple sclerosis, (2) musculoskeletal conditions affecting balance (osteoarthritis of the lower limb, recent lower limb surgery), (3) severe visual impairments uncorrected by lenses, (4) vestibular dysfunction or inner ear pathology, and (5) use of medications known to affect balance, such as sedatives or muscle relaxants. Participants meeting the inclusion criteria were invited to participate in the study during their routine endocrinology or rehabilitation clinic visits. A structured screening interview, clinical examination, and review of medical records were conducted to confirm eligibility. Proprioceptive measurements were performed by the same physiotherapist to ensure intra-rater reliability. The examiner was trained in the use of dual inclinometry and followed a standardized protocol. Peripheral neuropathy assessments using the TCSS were conducted by two independent neurologists trained in the scale, with diagnosis confirmed by nerve conduction studies. Any discrepancies in scoring were resolved through consensus. All examiners conducting proprioception and posturography testing were blinded to group allocation to minimize observer bias.

### Sample size calculation

The sample size was calculated using G*Power software to ensure adequate statistical power for detecting differences in ankle proprioception and postural balance impairments in individuals with T2DM. Based on prior studies, a moderate-to-large effect size (0.50–0.75, Cohen’s *d*) was assumed, with a correlation coefficient of 0.4 between proprioceptive deficits and postural stability. To achieve a power of 0.80 with a significance level of 0.05, the required sample size was determined to be 66 participants.

### Variables

This study examined proprioceptive deficits and postural balance impairments in individuals with T2DM and healthy controls. The variables of interest were categorized into demographic and clinical characteristics, proprioceptive function, postural balance measures, and metabolic predictors. All assessments were conducted under standardized conditions by trained physiotherapists and endocrinologists, ensuring accuracy and reproducibility.

### Demographic and clinical variables

The demographic variables included age, sex, and body mass index (BMI), which were obtained through patient interviews and medical records. Clinical data included diabetes duration (years), HbA1c levels (%), fasting blood glucose (mg/dL), and blood pressure (mmHg). Peripheral neuropathy status was determined using the Toronto Clinical Scoring System (TCSS) and confirmed by nerve conduction studies (NCS). A TCSS score ≥ 6 indicated the presence of peripheral neuropathy. HbA1c and fasting glucose levels were measured using automated enzymatic assays, and blood pressure was recorded using a calibrated sphygmomanometer following standardized clinical guidelines.

### Assessment of ankle proprioceptive function

**FIGURE 1:**
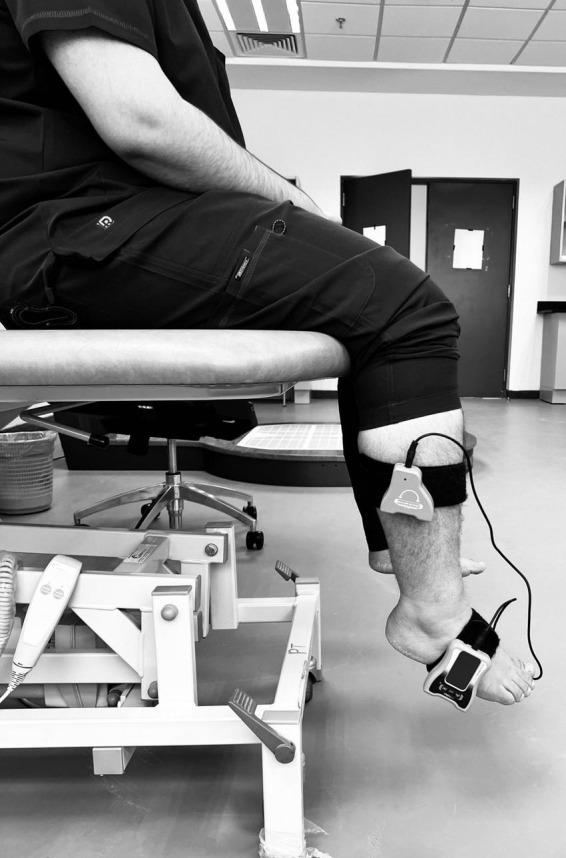
Assessment of ankle joint proprioception using dual digital inclinometry.

Ankle proprioception was assessed using a dual digital inclinometer, a validated tool for measuring joint position sense (JPS) errors in individuals with T2DM (Alahmari and Reddy, 2024). Participants were seated in a standardized position with knees flexed at 90 degrees and feet in a relaxed dangling posture to ensure a neutral baseline. The primary inclinometer was securely attached to the lateral aspect of the foot. In contrast, the secondary inclinometer was fixed to the lateral tibial shaft using a Velcro strap, allowing for precise measurement of ankle dorsiflexion and plantarflexion angles (Figure 1). Before testing, the inclinometer was calibrated to 0 degrees to establish a reference neutral position. During the assessment, participants were instructed to close their eyes to eliminate visual feedback, ensuring reliance solely on proprioceptive input. The examiner passively moved the participant’s ankle into a pre-determined dorsiflexion or plantarflexion angle (10° or 15° from neutral) and held the position for five seconds to allow for sensory encoding. The foot was then returned to the neutral position, and participants were instructed to actively reproduce the target angle to the best of their ability. Participants signaled when they believed they had reached the correct position, and the absolute angular error (difference between the target and reproduced angle) was recorded in degrees. Each movement direction was tested three times, and the average proprioceptive error was calculated. The procedure was performed bilaterally, with data from the dominant limb being used for primary analysis. Higher joint position sense error values indicated more significant proprioceptive impairment, reflecting reduced sensory feedback accuracy. Standardized instructions were given to all participants to ensure consistency and eliminate bias, and the same examiner conducted all measurements.

### Assessment of postural balance measures

**FIGURE 2:**
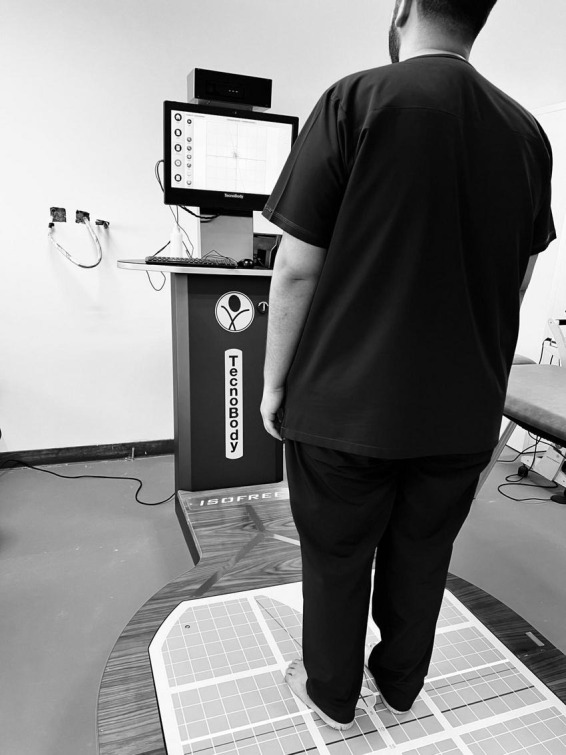
Postural stability evaluation using computerized posturography platform.

Postural balance was assessed using computerized posturography with a stabilometric force platform under eyes-closed conditions (Yin et al., 2021), which effectively isolates proprioceptive and vestibular contributions to balance control by eliminating visual input. Participants stood barefoot on the force platform in a natural upright stance, with feet positioned shoulder-width apart, ensuring consistency in postural alignment across all subjects. They were instructed to close their eyes and maintain balance for 30 s per trial, minimizing external visual cues that could influence postural stability (Figure 2). The force platform continuously recorded center of pressure (CoP) displacement in anteroposterior (AP) and mediolateral (ML) directions, capturing key balance parameters such as total postural sway area (cm^2^), sway velocity (cm/s), mean sway velocity (cm/s), and CoP trajectory length (cm). A greater postural sway area, increased CoP displacement, and higher sway velocity indicated reduced postural control and greater reliance on impaired somatosensory input. Each participant performed three consecutive trials, with the mean values used for statistical analysis to enhance measurement reliability. A 30-s rest interval was provided between trials to minimize fatigue effects.

### Data analysis

All statistical analyses were performed using SPSS version 24, with a significance level of *p* < 0.05. Data were assessed for normality using the Shapiro–Wilk test, confirming a normal distribution. Descriptive statistics were reported as mean ± standard deviation (SD). Independent *t*-tests were conducted to compare ankle proprioception and postural balance deficits between individuals with T2DM and healthy controls. The relationship between proprioception, postural instability, and glycemic control (HbA1c levels) was analyzed using Pearson’s correlation coefficient (*r*). A one-way ANOVA with *post hoc* Bonferroni correction was used to compare proprioceptive and balance impairments among subgroups, including individuals with and without peripheral neuropathy. Additionally, multiple linear regression analysis was performed to determine whether HbA1c levels, diabetes duration, BMI, and age were significant predictors of proprioceptive deficits and postural instability. A repeated-measures ANOVA was used to assess changes in postural sway under different sensory conditions in the posturography assessment. Assumptions for statistical tests were verified: normality was assessed using the Shapiro–Wilk test; homogeneity of variance was evaluated using Levene’s test; and multicollinearity in regression was checked using variance inflation factor (VIF) values (< 2.0). Data were screened for outliers using boxplots and standardized z-scores (threshold: ± 3.0). No extreme outliers were retained. Missing data were minimal (< 5%) and handled through pairwise deletion to preserve available cases. Statistical significance was defined as *p* < 0.05 for all tests.

## Results

Individuals with T2DM demonstrated significantly higher HbA1c levels, fasting blood glucose, and systolic blood pressure than healthy controls (*p* < 0.001, Table 1). No significant differences were observed in age, sex distribution, BMI, or diastolic blood pressure between the groups (*p* > 0.05). The mean diabetes duration among individuals with T2DM was 10.90 ± 3.99 years, and 42 out of 66 participants in this group exhibited peripheral neuropathy, highlighting a substantial burden of diabetes-related complications. No significant differences were observed between males and females in proprioceptive or postural balance parameters across both groups. As the groups were sex-matched and no interaction effect with sex was found, sex was not included as a stratification variable in further analyses.

**TABLE 1:** Demographic and clinical characteristics.

Variable	T2DM (*n* = 66)	Controls (*n* = 66)	*p*-value
Age (years)	57.35 ± 6.24	57.88 ± 6.47	0.634
Sex (male/female)	36/30	35/31	0.906
BMI (kg/m^2^)	28.70 ± 3.72	28.12 ± 4.47	0.426
Diabetes duration (years)	10.90 ± 3.99	NA	NA
HbA1c (%)	7.97 ± 1.07	5.29 ± 0.61	< 0.001
Fasting blood glucose (mg/dL)	139.35 ± 25.76	93.52 ± 11.79	< 0.001
Systolic blood pressure (mmHg)	133.38 ± 13.66	123.33 ± 10.72	< 0.001
Diastolic blood pressure (mmHg)	81.72 ± 8.24	79.21 ± 6.41	0.055
Peripheral neuropathy (yes/no)	42/24	NA	NA

BMI, body mass index; HbA1c, glycated hemoglobin; FBG, fasting blood glucose; SBP, systolic blood pressure; DBP, diastolic blood pressure; NA, not applicable; T2DM, type 2 diabetes mellitus; mmHg, millimeters of mercury.

Individuals with T2DM exhibited significantly greater proprioceptive deficits and postural instability than healthy controls (*p* < 0.001, Table 2). Ankle dorsiflexion and plantarflexion errors were markedly higher in the T2DM group, indicating impaired proprioception. Postural sway, sway velocity, sway area, and mean sway velocity were all significantly increased, reflecting reduced postural stability. Anteroposterior and mediolateral sway were also significantly greater, suggesting a compromised ability to maintain balance. Large effect sizes (Cohen’s *d* > 1.0) for most variables indicate substantial differences between the proprioceptive and postural control groups.

**TABLE 2:** Comparison of proprioception and postural stability between T2DM and healthy controls.

Variable	T2DM (*n* = 66)	Controls (*n* = 66)	Mean difference (95% CI)	*p*-value	Effect size (Cohen’s *d*)
Ankle dorsiflexion error (°)	5.03 ± 1.00	3.10 ± 0.87	1.93 (1.61 to 2.26)	< 0.001	2.055
Ankle plantarflexion error (°)	7.46 ± 1.18	4.96 ± 1.26	2.50 (2.08 to 2.92)	< 0.001	2.034
Postural sway (cm^2^)	178.67 ± 36.38	141.63 ± 29.13	37.04 (25.71 to 48.37)	< 0.001	1.115
Sway velocity (cm/s)	5.33 ± 1.14	3.98 ± 1.08	1.36 (0.98 to 1.74)	< 0.001	1.214
Sway area (cm^2^)	342.82 ± 45.50	280.06 ± 43.57	62.76 (47.44 to 78.07)	< 0.001	1.398
Mean sway velocity (cm/s)	6.25 ± 1.00	4.85 ± 1.03	1.40 (1.05 to 1.75)	< 0.001	1.372
Anteroposterior (AP) sway (cm)	2.89 ± 0.52	1.91 ± 0.44	0.98 (0.81 to 1.15)	< 0.001	2.008
Mediolateral (ML) sway (cm)	2.07 ± 0.52	1.45 ± 0.41	0.62 (0.46 to 0.79)	< 0.001	1.324

T2DM, type 2 diabetes mellitus; cm^2^, square centimeters; cm/s, centimeters per second; s, seconds; AP, anteroposterior; ML, mediolateral; CI, confidence interval.

**FIGURE 3:**
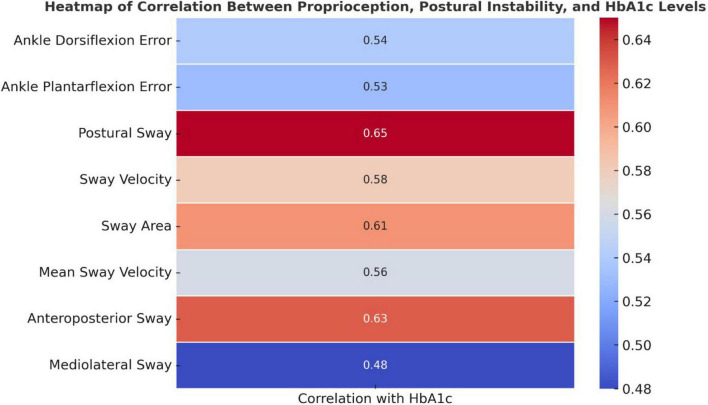
Heatmap of correlation between proprioception, postural instability, and HbA1c levels.

Higher HbA1c levels were significantly associated with increased proprioception errors and postural instability (*p* < 0.05, Figure 3). Ankle dorsiflexion and plantarflexion errors showed moderate positive correlations with HbA1c (*r* = 0.54 and 0.53, respectively), suggesting that worsening glycemic control is linked to impaired proprioception. Stronger correlations were observed for postural sway, sway velocity, sway area, and anteroposterior sway (*r* = 0.56–0.65), indicating a notable decline in postural stability with higher HbA1c levels.

Diabetic individuals with peripheral neuropathy exhibited significantly greater proprioceptive deficits and postural instability compared to those without neuropathy (*p* < 0.05, Table 3). Ankle dorsiflexion and plantarflexion errors were notably higher in the neuropathy group, indicating impaired proprioceptive accuracy. Postural stability measures, including postural sway, sway velocity, sway area, and mean sway velocity, were significantly worse in individuals with neuropathy, with large effect sizes (Cohen’s *d* > 0.9), reflecting substantial balance impairment. Anteroposterior and mediolateral sway were also significantly increased, suggesting a progressive decline in postural control with neuropathy severity. These findings highlight the detrimental impact of diabetic neuropathy on proprioceptive function and balance regulation.

**TABLE 3:** Comparison between diabetic individuals with and without peripheral neuropathy.

Variable	Neuropathy (*n* = 42) mean ± SD	No neuropathy (*n* = 24) mean ± SD	Mean difference (95% CI)	*p*-value	Effect size (Cohen’s *d*)
Ankle dorsiflexion error (°)	5.48 ± 0.98	4.72 ± 1.12	−0.76 (−1.18 to −0.34)	0.002	0.72
Ankle plantarflexion error (°)	7.98 ± 1.10	6.08 ± 1.25	−0.90 (−1.35 to −0.45)	0.001	0.83
Postural sway (cm^2^)	192.54 ± 38.71	162.35 ± 32.69	30.19 (15.12 to 45.26)	< 0.001	1.02
Sway velocity (cm/s)	5.68 ± 1.21	4.85 ± 1.03	0.83 (0.42 to 1.24)	< 0.001	0.91
Sway area (cm^2^)	358.34 ± 47.82	318.22 ± 42.15	40.12 (22.47 to 57.77)	< 0.001	1.08
Mean sway velocity (cm/s)	6.58 ± 1.11	5.98 ± 1.02	0.60 (0.23 to 0.97)	0.003	0.68
Anteroposterior (AP) sway (cm)	3.01 ± 0.55	2.74 ± 0.50	0.27 (0.08 to 0.46)	0.009	0.55
Mediolateral (ML) sway (cm)	2.21 ± 0.50	1.88 ± 0.47	0.33 (0.15 to 0.51)	0.001	0.7

SD, standard deviation; CI, confidence interval; AP, anteroposterior; ML, mediolateral; cm, centimeters; cm^2^, square centimeters; cm/s, centimeters per second;°, degrees; *p*-value, probability value; Cohen’s *d*, effect size (Cohen’s *d*).

Higher HbA1c levels, longer diabetes duration, increased BMI, and older age were significant predictors of proprioceptive deficits and postural instability, with HbA1c showing the strongest association (β = 0.42, *p* < 0.001, Table 4). Diabetes duration was also a significant predictor (β = 0.38, *p* = 0.001), indicating that longer disease duration contributes to impaired balance and proprioception. BMI and age demonstrated moderate effects (β = 0.31 and 0.27, respectively, *p* < 0.05), suggesting that metabolic and age-related factors influence postural control. The regression model explained a substantial proportion of variance in proprioceptive deficits (adjusted *R*^2^ = 0.29–0.48), with HbA1c emerging as the most influential factor in balance impairment.

**TABLE 4:** Regression analysis predicting proprioceptive deficits and postural stability.

Predictor variable	β Coefficient (95% CI)	Standard error	*p*-value	Adjusted *R*^2^	F-statistic
HbA1c (%)	0.42 (0.28 to 0.56)	0.07	< 0.001	0.48	15.74
Diabetes duration (years)	0.38 (0.24 to 0.52)	0.06	0.001	0.42	12.39
BMI (kg/m^2^)	0.31 (0.18 to 0.44)	0.06	0.004	0.35	9.58
Age (years)	0.27 (0.15 to 0.39)	0.05	0.007	0.29	7.83

HbA1c, glycated hemoglobin; BMI, body mass index; CI, confidence interval; *p*-value, probability value; β, standardized beta coefficient; *R*^2^, adjusted R-squared; F-statistic, F-test for overall model significance.

## Discussion

This study aimed to compare ankle joint proprioception and postural balance deficits between individuals with T2DM and healthy controls, while also assessing the impact of proprioceptive impairments on postural stability and their relationship with glycemic control. The results demonstrated that individuals with T2DM exhibited significantly greater proprioceptive deficits and postural instability compared to healthy controls, with notable impairments in ankle movement accuracy, postural sway, and stability measures. Furthermore, poorer glycemic control was associated with greater proprioceptive errors and postural instability, indicating that elevated blood glucose levels may contribute to worsening balance function. The subgroup analysis revealed that diabetic individuals with peripheral neuropathy had significantly worse proprioception and balance performance than those without neuropathy, highlighting the role of neuropathy in progressive sensorimotor decline. Additionally, regression analysis identified glycemic control, diabetes duration, BMI, and age as significant predictors of proprioceptive and balance impairments, with glycemic control emerging as the strongest determinant. These findings emphasize the detrimental effects of diabetes on proprioception and postural stability and underscore the importance of early screening and targeted interventions to reduce fall risk and enhance functional stability in individuals with T2DM.

The observed deficits in proprioception and postural stability among individuals with T2DM can be attributed to multiple physiological mechanisms associated with diabetes-induced sensorimotor dysfunction (Tian et al., 2024). Peripheral neuropathy, a common complication of diabetes, leads to deterioration of mechanoreceptors in the ankle joint, reducing the ability to detect and respond to positional changes, increasing proprioceptive errors (Tian et al., 2024). Additionally, hyperglycemia-induced oxidative stress and microvascular damage impair nerve conduction velocity, compromising somatosensory feedback and motor control (Reeves et al., 2021). The significant increase in postural sway and instability measures suggests that individuals with T2DM rely more on visual and vestibular input for balance, as their somatosensory input is compromised (Kaur et al., 2023). The correlation between higher HbA1c levels and greater proprioceptive errors indicates that poor glycemic control exacerbates sensorimotor deficits, likely due to chronic hyperglycemia-mediated neurodegeneration (Komalasari et al., 2024). Moreover, secondary to diabetes-related metabolic changes, muscle weakness and altered tendon stiffness may contribute to reduced postural stability and delayed motor responses, further impairing balance regulation in this population (Al-Modhefer, 2021). The findings of this study are consistent with previous research demonstrating significant proprioceptive and postural deficits in individuals with T2DM (Reeves et al., 2021; Reisi et al., 2024). Reeves et al. (2021) reported that diabetic neuropathy leads to altered proprioceptive acuity and increased postural instability, particularly in challenging balance conditions (Reeves et al., 2021). Similarly, Reisi et al. (2024) found that diabetic individuals exhibited greater postural sway and reduced sway control compared to healthy controls, highlighting the role of somatosensory impairment in balance dysfunction. Furthermore, Alahmari and Reddy (2024) identified a strong association between glycemic control and proprioceptive deficits, reinforcing the current study’s findings that higher HbA1c levels correlate with increased proprioceptive errors and instability. The significant impact of neuropathy on balance control is also supported by Alissa et al. (2024), who demonstrated that diabetic individuals with neuropathy had worse proprioceptive accuracy and greater fall risk than those without neuropathy (Alissa et al., 2024). These findings further substantiate the detrimental effects of diabetes on sensorimotor function and postural regulation, emphasizing the importance of early intervention strategies, including proprioceptive training and glycemic management, to mitigate balance impairments and reduce fall risk in individuals with T2DM (Alissa et al., 2024).

The significant association between elevated HbA1c levels and increased proprioceptive errors and postural instability in individuals with type 2 diabetes mellitus (T2DM) can be explained by multiple pathophysiological mechanisms linked to chronic hyperglycemia (ALMohiza et al., 2023; Alahmari and Reddy, 2024). Long-term elevated blood glucose leads to structural and functional damage in peripheral nerves—especially the large afferent fibers—thereby delaying sensory transmission from the ankle to the central nervous system and increasing errors in joint position sense (Alshahrani et al., 2024; ALMohiza et al., 2023; Alahmari and Reddy, 2024). This sensory degradation is compounded by microvascular damage, oxidative stress, demyelination, and axonal degeneration, all of which impair both proprioceptive input and motor output (Alshahrani et al., 2024). Consequently, higher HbA1c levels strongly correlate with postural sway, indicating that impaired proprioception translates to compromised balance control (Rasmussen et al., 2022). In addition, diabetes-related muscle weakness and altered tendon stiffness further exacerbate balance impairments. Multiple studies support these findings, including those by ALMohiza et al. (2023), Reeves et al. (2021), and Malwanage et al. (2024), who reported consistent links between poor glycemic control, impaired proprioception, and postural instability, thereby underscoring the importance of maintaining glycemic control to preserve neuromuscular integrity.

Furthermore, individuals with diabetic peripheral neuropathy (DPN) exhibit significantly greater proprioceptive deficits and postural instability compared to those without neuropathy, due to the progressive degeneration of large-diameter myelinated fibers responsible for accurate sensory feedback (Reeves et al., 2021). This degeneration disrupts the detection of joint position and impairs muscle spindle and cutaneous mechanoreceptor functions, leading to increased proprioceptive errors and compensatory dependence on visual and vestibular inputs (Reeves et al., 2021). Longer diabetes duration and higher HbA1c levels further accelerate these deficits (Treleaven, 2024). Research by Reeves et al. (2021), Lopatin et al. (2025), and Khan et al. (2021), supports these observations, showing that neuropathic individuals experience marked balance impairments and greater fall risk. Orlando et al. (2022) additionally highlight the progressive decline in joint position sense in this population. These findings emphasize the need for early detection of neuropathy, strict glycemic management, and targeted balance interventions to mitigate instability and reduce fall risk in individuals with DPN.

### Clinical significance

The clinical significance of this study lies in its identification of proprioceptive deficits and postural instability as key impairments in individuals with T2DM, particularly those with peripheral neuropathy and poor glycemic control. The findings highlight the strong association between higher HbA1c levels and greater proprioceptive errors and postural sway, emphasizing the need for strict glycemic management to mitigate sensorimotor dysfunction. Additionally, the observed greater balance impairments in neuropathic individuals reinforce the necessity for early screening and targeted interventions to prevent progressive postural instability and reduce fall risk, a major cause of morbidity in this population. Given the significant impact of diabetes duration, BMI, and age on proprioceptive function, a multifactorial rehabilitation approach incorporating neuromuscular training, proprioceptive exercises, and weight management strategies is warranted to improve functional stability and mobility. These findings provide critical evidence for integrating proprioception-based balance assessments into routine clinical evaluations for individuals with T2DM, enabling early identification of high-risk patients and the implementation of preventative measures to enhance postural control and overall quality of life.

### Limitations and future directions

Despite the significant findings, this study has several limitations that should be acknowledged. First, while the study establishes a strong association between HbA1c levels, proprioceptive deficits, and postural instability, its cross-sectional design prevents causal inferences, necessitating longitudinal studies to assess the progression of these impairments over time. Second, the study did not account for other potential confounding factors, such as medication use, physical activity levels, or comorbid conditions (retinopathy or vestibular dysfunction), which may also influence balance and proprioception. Additionally, peripheral neuropathy severity was not categorized into mild, moderate, or severe, limiting the ability to evaluate progressive proprioceptive decline across different stages of neuropathy. The reliance on dual inclinometry and posturography, while effective, may not fully capture real-world postural challenges, highlighting the need for ecologically valid balance assessments, such as functional gait analysis or dynamic perturbation tests. Additionally, while the study controlled for major neurological, vestibular, and musculoskeletal confounders, it did not collect data on fundus examination, lipid profiles, smoking history, or detailed cardiovascular comorbidities. However, the posturography protocol was conducted under eyes-closed conditions to eliminate visual input, and blood pressure was assessed and reported. The exclusion of additional systemic clinical data was deliberate to focus on proprioceptive and sensorimotor mechanisms; nonetheless, future research should incorporate broader clinical variables to enhance external validity. Additionally, while glycemic control was assessed using HbA1c and fasting blood glucose—standard markers with high clinical relevance—we recognize that this represents a limited view of metabolic regulation. Other indices such as postprandial glucose, glycemic variability, and composite clinical scoring systems were not included. Future studies should consider incorporating more comprehensive glycemic profiles to better contextualize the relationship between metabolic control and sensorimotor dysfunction. Although individuals with clinically diagnosed neurological, vestibular, or visual disorders were excluded, we acknowledge the possibility that subclinical microvascular complications—such as early-stage retinopathy or cerebrovascular changes—may have been present in participants with ≥ 5 years of diabetes duration. These could exert subtle effects on sensorimotor function. However, the use of eyes-closed posturography minimized the influence of visual input on balance assessment, and the exclusion criteria were rigorously applied to reduce confounding. Future studies should consider neuroimaging and ophthalmological screening to comprehensively exclude such subclinical variables. Future research should focus on longitudinal studies to explore the trajectory of proprioceptive decline in T2DM, the role of targeted proprioceptive training interventions, and the integration of advanced sensorimotor rehabilitation techniques to improve balance and fall prevention strategies in this population.

## Conclusion

This study demonstrates that individuals with T2DM exhibit significantly greater proprioceptive deficits and postural instability compared to healthy controls, with impairments being more pronounced in those with peripheral neuropathy and poor glycemic control. Higher HbA1c levels were strongly associated with increased proprioceptive errors and postural sway, indicating that poor glycemic regulation contributes to sensorimotor dysfunction. Additionally, diabetic individuals with peripheral neuropathy showed significantly worse proprioceptive accuracy and postural control, reinforcing the impact of neuropathy-related afferent dysfunction on balance regulation. Regression analysis identified HbA1c, diabetes duration, BMI, and age as significant predictors of proprioceptive and postural impairments, with glycemic control emerging as the strongest factor influencing balance stability. These findings emphasize the importance of early proprioceptive assessments, neuropathy screening, and targeted interventions to mitigate fall risk and improve postural control in individuals with T2DM.

## Data Availability

The datasets presented in this study can be found in online repositories. The names of the repository/repositories and accession number(s) can be found in this article/Supplementary material.
